# GUANinE v1.1 reveals complementarity of supervised and genomic language models

**DOI:** 10.1093/bioinformatics/btag427

**Published:** 2026-08-21

**Authors:** Eyes S Robson, Nilah M Ioannidis

**Affiliations:** Center for Computational Biology, UC Berkeley, Berkeley, CA 94720, United States; Department of Electrical Engineering and Computer Sciences, UC Berkeley, Berkeley, CA 94720, United States; Department of Applied Mathematics, UC Santa Cruz, Santa Cruz, CA 95064, United States; Chan Zuckerberg Biohub, San Francisco, CA 94158, United States

## Abstract

*Summary:* There has been much debate about the benefits of supervised versus unsupervised learning on genomes. Determining which is better in what contexts requires developing comprehensive benchmarks spanning functional and evolutionary tasks. Importantly, such benchmarks need large sample sizes to enable well-powered ranking of models. Having developed and applied such a benchmark here (GUANinE v1.1), we conclusively demonstrate each paradigm offers key advantages and outperforms on certain tasks. In accordance with training, supervised sequence-to-function models exhibit strong performance when annotating functional states characterized by chromatin accessibility or histone marks, while self-supervised language models outperform on evolutionary conservation. Our hundreds of new evaluations in this v1.1 expansion provide evidence for a tradeoff between input context size and model parameter count for a fixed compute budget, which we depict with new metrics such as kiloparameters/base pair. We also construct two new large-scale variant interpretation tasks in v1.1: cadd-snv measuring deleteriousness, and clinvar-snv measuring clinical pathogenicity. We find that conservation scores, and by extension, genomic language models, predict deleteriousness well, but successfully translating deleteriousness predictions to pathogenicity remains challenging. GUANinE v1.1 newly evaluates dozens of pretrained genomic models, and we conclude that moderate-context hybrid or post-trained language models may define the next era of machine learning in genomics.

## Introduction

Benchmarks have a long history in computing from high-performance computing to computer vision (Lecun *et al.* 1998) and can systematically assess model performance across field-encompassing tasks. Designing a benchmark involves careful consideration of tasks, data provenance, and *construct validity*, which measures the claim that a task represents an underlying research concept (Raji *et al.* 2021). For example, a DNA sequence-to-function task designed to evaluate chromatin accessibility predictions based on a single ATAC-seq experiment yields a different task from one constructed in a different cell line or using a different type of accessibility data, for example, FAIRE-seq.

Several benchmarks have been developed in genomics to evaluate supervised sequence-to-function models and genomic language models (Marin *et al.* 2024, Patel *et al.* 2024). Additionally, substantial benchmark*ing* has been undertaken in the field, both accompanying new models (Avsec *et al.* 2026) and in standalone or competition-style research (Huang *et al.* 2023, Karollus *et al.* 2023, Sasse *et al.* 2023, Tang *et al.* 2025).

Our existing benchmark, GUANinE v1.0 (Robson and Ioannidis 2023), included tasks designed to model tissue-agnostic human-genome functional signal aggregated across many cell lines, rather than relying on individual tissue-specific measurements. Intended to increase its broad applicability and long-term relevance, this layer of abstraction gives GUANinE independence from any one experiment. However, the v1.0 release of GUANinE was dedicated to task verification, rather than seeking to widely evaluate models in the field. In this new v1.1 release, we now:

1. Newly report performances of dozens of recent state-of-the-art pretrained supervised and genomic language models (gLMs) with over 9 zetta-FLOPs of compute.
2. Create and evalute two new *variant effects* tasks: cadd-snv for deleteriousness and clinvar-snv for pathogenicity.

A unique feature of GUANinE, which we retain in v1.1, is its precision achieved through unrivaled sample sizes. Large-sample settings combined with linear evaluation, sometimes referred to as ‘probing’, eliminate numerous issues that arise when fine-tuning on small sample sizes. For example, it removes the confounding factor of fine-tuning hyperparameters, reducing the potential ‘human error’ of tweaking an unfamiliar model, and because weights are frozen, high-dimensional models are not disadvantaged by statistical inefficiency (Bickel 1984). As a result, GUANinE resembles the asymptotic suitability of pretrained models to tasks, subject only to construct validity.

The previously described tasks from GUANinE v1.0 span three domains: regulatory element annotations, sequence alignment conservation, and synthetic yeast promoters. Each domain has two tasks to ensure robustness. See the Supplementary Material for a detailed description of existing tasks, briefly enumerated here:

1. dnase-propensity (Regulatory, hg38) – tissue-agnostic chromatin accessibility aggregated from tissue-specific DNase-seq experiments.
2. ccre-propensity (Regulatory, hg38) – cis-regulatory element inference, based on accessible histone marks/CTCF.
3. conservation30 (Evolutionary, hg38) – mammalian conservation annotation.
4. conservation100 (Evolutionary, hg38) – vertebrate conservation annotation.
5. gpra-c (Promoter Expression, yeast) – dual-reporter assay.
6. gpra-d (Promoter Expression, yeast) – as gpra-c.

## Methods and datasets

### Newly evaluated models

We evaluate a wide range of models on the GUANinE benchmark tasks. While these models are not an exhaustive list of sequence-to-function and genomic language models (gLMs) in the field, we attempted to include a diverse set of models. We preclude some eclipsed by their original developers (e.g. Nucleotide Transformer v1). The models with newly reported results are:

1. Two GM12876 ChromBPNet models, Folds 0 and 3, trained on ATAC-seq. We also evaluate raw input ENCFF180XQC.
2. DNABERT-2 gLM.
3. PhyloGPN phylogenetic-loss gLM.
4. All four Nucleotide Transformer v2 (NTv2) gLMs.
5. Three Nucleotide Transformer v3 (NTv3) gLMs and two supervised derivatives.
6. The ensemble-distilled Enigma model.
7. Six GENA-LM gLMs, plus one version fine-tuned on Enformer’s training data.
8. The contrastive dna2vec of ‘Embed-Search-Align’.
9. The supervised CNN Sei.
10. Two Mistral-DNA and two ModernBERT models.
11. Two Caduceus genomic language models, PS and PH.
12. Two of the Evo2 gLMs, including a second evaluation of Evo2_7b at a higher-context size.
13. Two of the AI Digital Organism (AIDO) gLMs.

For the newly released cadd-snv and clinvar-snv tasks, we include a handful of additional variant effects models:

1. CADD v1.7, an annotation-based supervised deleteriousness model trained with simulation-augmented data.
2. Pangolin, an RNA-splicing model.
3. GPN-MSA, a conservation-centric MSA-to-function model.
4. AlphaGenome, ands its framework for variant interpretation.
5. Variant Effect Predictor (VEP) build 115 as a baseline.

### Newly constructed tasks

The cadd-snv task requires models to separate frequent or evolutionarily inferred fixed variants from ultra-rare variation—a challenging task that approximates deleteriousness. While the original authors of varCADD caution against over-optimizing for deleteriousness (Nazaretyan *et al.* 2025), as doing so can detract from *pathogenicity* generalization (e.g. to clinvar-snv), we posit that the population-derived varCADD dataset is still useful for evaluating models’ understanding of variation, and it has the benefit of tremendous size (Table 1) to enable robust model comparisons. A significant portion of cadd-snv is intergenic (potentially regulatory) variants; figures detailing the dataset are available in the Supplement.

**Table 1:** Summary statistics of new variant tasks.

Task	Proxy deleteriousness	Pathogenic effects
	cadd-snv	clinvar-snv
Training set	76 M	–
1% Training set	760 K	–
Dev set	1.03 M	9.2 K
Test set	1.04 M	12.0 K
Split	Dev21/Test22	Dev21/Test22

Genome	hg38	hg38
Target	Boolean	Boolean
Output range	0–1	0–1
Source	Nazaretyan *et al.* (2025)	Landrum *et al.* (2014)

cadd-snv asks models to distinguish between proxy deleterious, singletons in gnomAD v3, and proxy benign variants, which are simulated or frequently observed [known as hfs (Nazaretyan *et al.* 2025)]. clinvar-snv asks models to distinguish between benign and pathogenic variants and predominantly consists of benign intronic and pathogenic splice site variants. While imbalanced, it is representative of noncoding variants in the ClinVar database.

The second new task, clinvar-snv, features no training set and is the smallest task yet in GUANinE, totaling just fewer than 12 000 variants. However, its count of nearly 6000 pathogenic variants is still an order of magnitude larger than a comparable benchmark (Benegas *et al.* 2025). We note that the underlying *construct validity* of clinvar-snv is not general pathogenicity; rather, it is a well-controlled subset of the splicing-variant-rich ClinVar database, see Fig. S3 for details.

### Evaluation methods

We perform feature extraction on frozen models and fit a ‘few-shot’ linear probe to these extracted outputs or embeddings (Pedregosa *et al.* 2011, Kathail *et al.* 2025). The key to linear evaluation is identifying the proper regularization strength, which we accomplish by sweeping over a wide range of parameter values during ‘training’, namely $C\in [0.01,0.03,\ldots ,10^{5},3\times 10^{5}]$ for Ridge regression. For logistic regression, which is inverted, we use $\alpha \in [10^{-6},3\times 10^{-6},\ldots ,0.3,1]$. The best performing model on the development set was selected for test set inference. Due to high dimensionality, Sei required higher regularization.

For feature extraction on the cadd-snv task, we use the log_2_ fold change of model outputs, except gLMs, where we use the contrast of alt—ref last-layer embeddings. Other than the zero-shot performances of phyloGPN and GPN-MSA, we do not use gLM logits.

For other tasks, we use last-layer embeddings for all gLMs (except Evo2), and we primarily use outputs from supervised models, excepting the ChromBPNet models, where we use embeddings instead of one-dimensional outputs. As previously reported (Robson and Ioannidis 2025), supervised models like Enformer often have better general performance at the penultimate layer, but we do not believe this to be the case for top-heavy models like DeepSEA or Sei.

## Results

Abbreviated findings for newly evaluated models are presented in Table 2. The full tabulation with models discussed in less detail can be seen in Table S5. New variant task results are presented separately in Tables 3 and 4.

**Table 2:** Abbreviated new results on existing tasks.

				dnase-	ccre-	**Conservation**		gpra-c	gpra-d
				**Propensity**		30	100		
Model	Reg.	Eval.	Context	ρ	ρ	ρ	ρ	ρ	ρ
GC-content	B		511±1	20.55	12.99	38.13	39.58	52.97	56.98
5-mer LinSVR	B	F	511±1	36.30	26.37	49.35	45.05	69.43	73.65
5-mer *k*NN	B	F	511±1	31.99	23.36	52.58	44.54	58.56	62.11
⋆ T5		F	511±1	33.05	27.46	52.56	44.05	**84.67**	**88.59**
hgT5-8195	U + S	F	511±1	56.58	36.10	59.41	46.16	84.08	87.66
DeepSEA	S	X	=1 k	34.69	23.22	49.62	43.40	68.66	72.97
Enformer	S	X	≈197 k	64.92	55.38	74.44	62.23	70.63	74.13
Enformer (Pre-output)	S	X	≈197 k	68.08	55.08	74.68	62.42	70.70	74.20
Borzoi	S	X	≈524 k	70.69	57.69	71.55	59.69	71.53	75.18

ENCFF180XQC	B		511±1	24.16	34.85	4.09	6.46	–	–
ChromBPNet-ATAC-F0	S	X	= 2114	41.49	40.42	47.78	41.49	71.09	74.93
ChromBPNet-ATAC-F3	S	X	= 2114	40.55	40.47	47.41	40.32	70.62	74.24
Enigma-Distilled	S	X	≈524 k	65.76	51.76	63.02	45.81	67.95	71.45
Sei	S	X	= 4 k	70.47	56.35	63.19	51.58	68.85	72.18
AIDO.DNA_300m	U	X	=4 k	42.66	35.38	60.99	51.50	73.52	77.25
ESA-dna2vec	U	X	= 500	26.00	17.43	44.09	39.79	59.12	62.79
Caduceus-PS	U	X	≈131 k	42.97	41.32	74.37	62.38	72.44	76.03
DNABERT-2	U	X	∼12 k	41.66	32.75	60.59	50.36	69.88	73.91
GENA-LM-bert-base-t2t	U	X	∼4.5 k	42.82	34.87	66.65	55.96	71.07	74.81
GENA-LM-bert-base-t2t-enf	U + S	X	∼4.5 k	54.29	48.74	68.18	56.54	70.63	74.21
GENA-LM-bert-large-t2t	U	X	∼4.5 k	44.43	36.17	71.63	59.88	71.51	75.28
GENA-LM-bigbird-base-t2t	U	X	∼36 k	44.10	37.33	73.60	61.51	71.37	75.05
Evo2_1B_base	U	X	≈8.2 k	41.79	40.23	68.49	55.22	72.65	76.38
Evo2_7B_base	U	X	≈8.2 k	46.90	45.52	74.07	61.89	75.50	79.07
Evo2_7B	U	X	≈32.8 k	47.16	47.09	**76.71**	**65.31**	†	†
Mistral-DNA-422m-hg38	U	X	∼10 k	39.22	36.06	60.54	50.73	69.43	73.17
NTv2-100m	U	X	∼12 k	42.08	35.72	69.23	57.93	71.37	75.11
NTv2-500m	U	X	∼12 k	46.61	38.20	75.58	63.52	72.67	76.58
NTv3-100m-pre	U	X	≈32.8 k	42.73	34.62	65.77	52.94	73.01	76.81
NTv3-100m-post	U + S	X	≈32.8 k	72.50	59.31	72.01	59.63	70.00	73.59
NTv3-650m-post	U + S	X	≈32.8 k	**75.29**	**59.97**	73.27	60.59	71.77	75.41
PhyloGPN	U	X	= 481	40.48	18.40	53.22	43.13	66.87	70.74

Previously reported performances are listed in blue. Models are grouped by pretraining regime and alphabetically by family. The best reported score is **bolded** per task. We underline the second best reported score. The ⋆ T5 is a fully supervised neural baseline. Models evaluated only on variant effect tasks appear in Tables 3 and 4.

Regimes: B → non-neural Baseline ∣ M → MSA-based method ∣ S → Supervised pretraining ∣ U → Unsupervised pretraining ∣∣  †→ Computationally infeasible Evaluation: F → Fine-tuned ∣ X → 1% eval ∣ ‘ ’ → zero-shot ∣∣ Context: = → exactly ∣  ≈  → rounded to ∣  ∼  → on the order of.

**Table 3:** Cadd-snv deleteriousness prediction performance is dominated by conservation scores, in particular phyloP-30way and the neural GPN-MSA.

				cadd-snv
Model	Reg.	Eval.	Context	ρ
GC-content	B		511±1	1.47
Naïve VEP 115 Conseq.	B	X	–	0.16
deltaSVM GM12878 DHS	B		19	7.61
GPN-MSA	M			**29.12**
GPN-MSA (Position)	M			26.65
phyloP-100way	M			23.28
phyloP-30way	M			28.72
CADD v1.7	S		–	18.15
CADD v1.7 (Position)	S		–	19.66
ChromBPNet-ATAC-F0	S	X	2114	17.29
ChromBPNet-ATAC-F3	S	X	2114	16.76
Enformer	S	X	≈197 k	17.10
Borzoi	S	X	≈524 k	20.99
Sei	S	X	=4 k	19.91
Evo2_1B_base	U	X	≈8.2 k	24.50
NTv2-100m	U	X	∼12 k	23.89
NTv2-250m	U	X	∼12 k	25.40
NTv2-500m	U	X	∼12 k	26.47
NTv3-8m-pre	U	X	≈32.8k	19.52
NTv3-100m-pre	U	X	≈32.8k	22.86
NTv3-100m-post	U	X	≈32.8k	22.14
PhyloGPN	U		= 481	9.80
PhyloGPN	U	X	= 481	23.60

Those same models underperform on pathogenicity prediction for variants in clinvar-snv (Table 4), showcasing the reason for the strategic *underfitting* to deleteriousness performed by the CADD developers to improve CADD’s transferability to pathogenicity.

**Table 4:** Clinvar-snv performance is dominated by splicing models and low-dimensional scorers like AlphaGenome’s.

			clinvar-snv
Model	Reg.	Context	ρ
GC-content	B	511±1	11.53
Naïve Conseq. Type	B	–	76.74
phyloP-30way	M		66.09
GPN-MSA	M		80.72
phyloP-100way	M		81.51
PhyloGPN	U		67.59
AlphaGenome-K562	S	≈1.05M	93.17
CADD v1.7 (Position)	S	–	83.68
CADD v1.7	S	–	84.64
Pangolin	S	≈10k	85.63
Sei-Aggr.	S	= 4k	50.85
Sei-Aggr. + Pangolin	S + S	≈10k	**95.13**

This scoring strategy, when applied to Sei as ‘Sei-Aggr.’ and combined with the splicing model Pangolin, outperforms even AlphaGenome. Note clinvar-snv has no training set, due to dataset size, so models are calibrated to the development set if they cannot zero-shot.

### Comparison of supervised and unsupervised models

**Figure 1:**
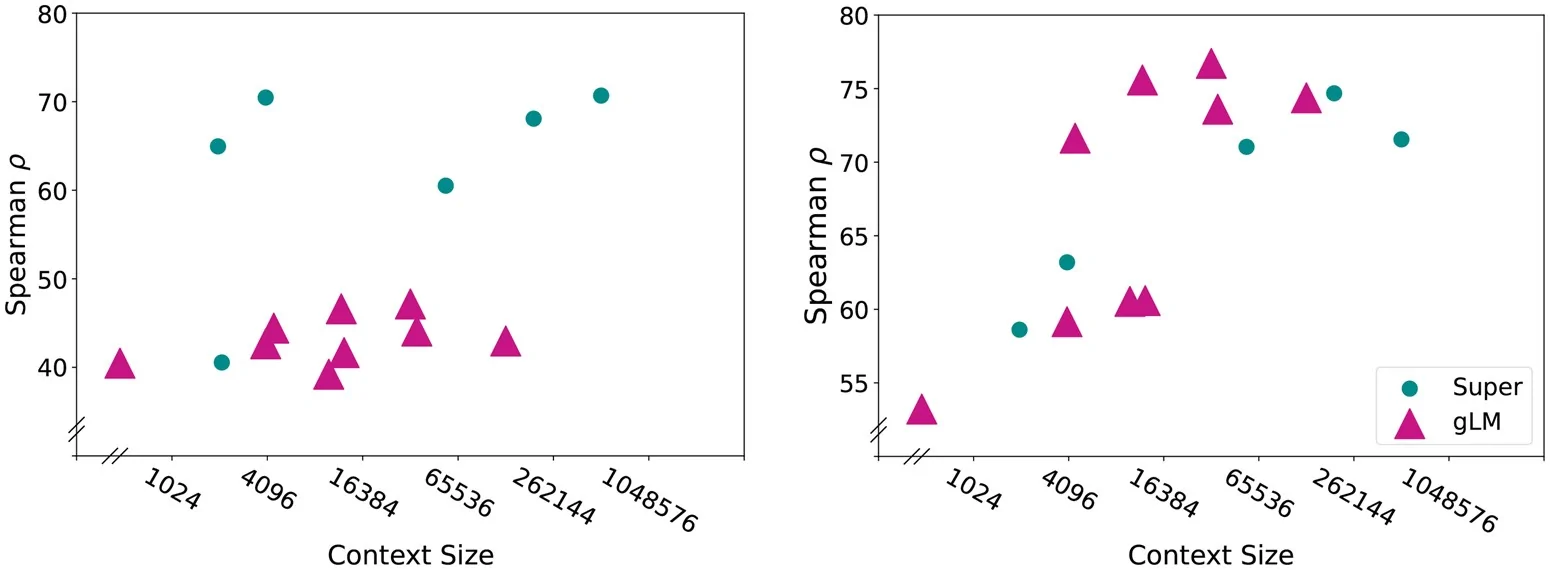
Scatterplots of performance on dnase-prop and cons30 as a function of context size and model type. Context size, while helpful, is only weakly predictive of performance compared to other factors. (Left) dnase-prop performance. Supervised models (blue dots) outperform for functional annotation, such as the accessibility task evaluated here, compared to unsupervised gLMs. (Right) cons30 performance. Unsupervised gLMs narrowly beat out supervised approaches, despite having less information during training.

First and foremost, our results show that in the few-shot regime, supervised methods like Borzoi, NTv3-post, and Sei outperform at annotating function relative to gLMs [dnase-propensity and ccre-propensity, Table 2; Fig. 1 (left)]. We believe this gap is due to the data-richness of supervised functional element annotations available in humans, the same data used to derive the labels in our functional evaluation sets. We also note that there does not appear to be a significant ‘test set leakage’ effect for models being evaluated on dnase-prop and ccre-prop, given the minor divergence of ChromBPNet-ATAC Fold 0, which sees test-set chromosomes 21 and 22 during training, and ChromBPNet-ATAC Fold 3, which does not.

As seen in Table 2 by the performance of NTv2, Evo2, and GENA-LM on cons30 and cons100, a clear advantage of gLMs over supervised-only approaches is their ability to capture sequence conservation information. While the gap on cons30 is small (Table 2; Fig. 1 (right)), these gLMs have been given objectively less supervision and are likely to further outperform if fine-tuned, for example, NTv3-post. We also point to the previously reported gains of hgT5-8195 (Robson and Ioannidis 2023)and to GENA-LM-bert-base-t2t-enformer on functional tasks.

### Scaling laws apply to gLMs

**Figure 2:**
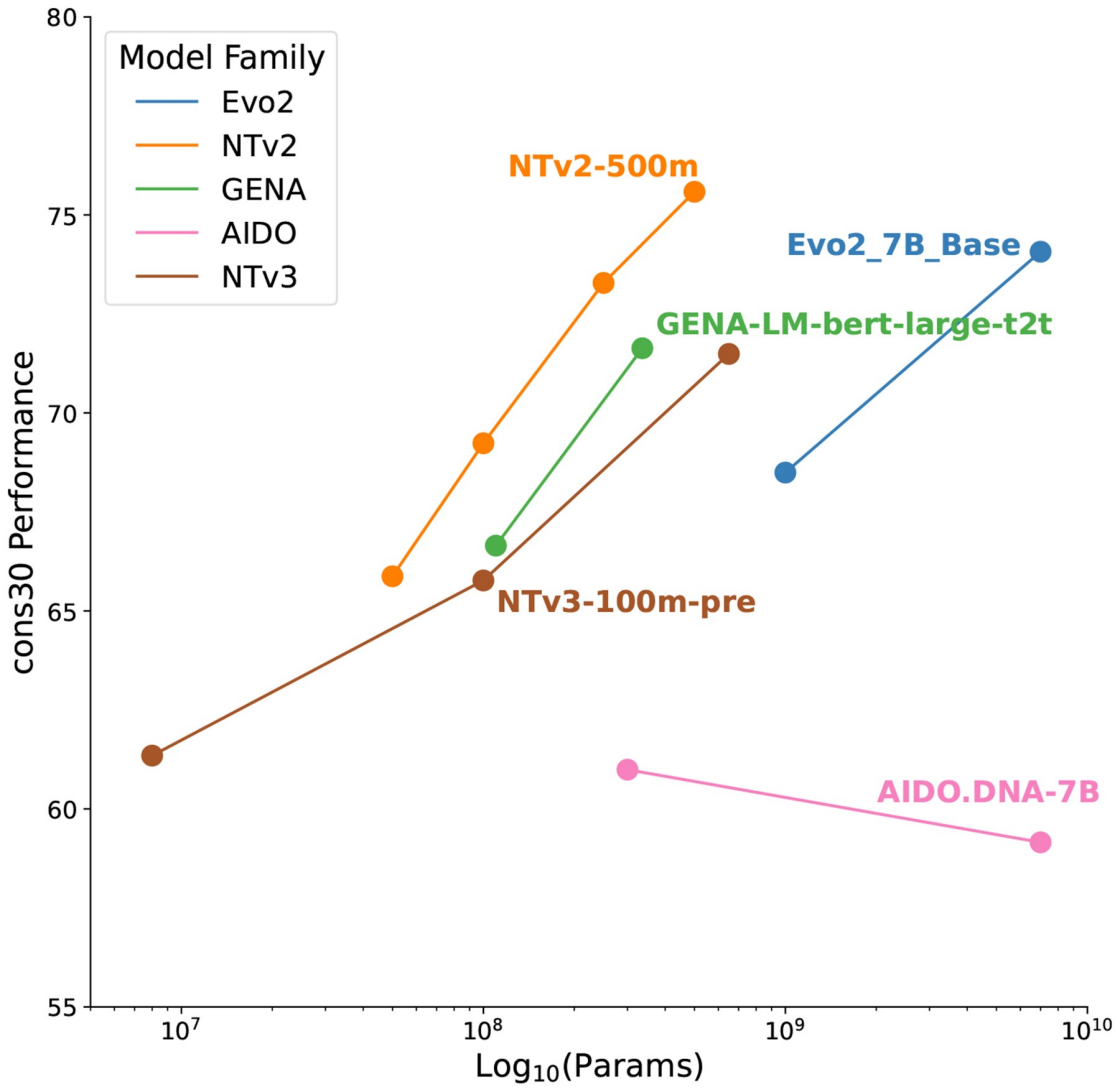
Scaling laws; scaling families. Conservation task performance demonstrates emergent *scaling laws* as a function of model parameter count, when conditioned on the same training corpus and architecture. Some model families, like Evo2 or GENA-LM, are less competitive than NTv2, but scaling helps them offset this gap. AIDO models, on the other hand, diverge in both starting point and scaling results.

On the topic of scaling laws (Kaplan *et al.* 2020), we confirm that larger models tend to improve downstream performance. We observe in Fig. 2 that different pretraining corpora and architectures start a model family at different locations on the scaling law frontier. Given the 300M and 7B AIDO models’ results, which do not follow the same trend as other families, we suspect but cannot confirm development flaws.

### More context ≠ better model

Next, we consider the trend of expanding input context (Linder *et al.* 2025, Avsec *et al.* 2026). For the tasks evaluated here, we note the performance of the 4 kbp-context Sei, which for functional annotation is nearly on par with the much larger 196 kbp-context Enformer and 524 kbp-context Borzoi models (dnase-prop and ccre-prop tasks in Table 2). We attribute Sei’s success to its parameter density, see ‘Digging into Sei’.

Similarly, lightweight, high-context architectures, such as the unsupervised Caduceus models, show promise, but the parameter-dense 500M Nucleotide Transformer v2 still achieves a higher performance on cons30 and cons100. Despite 65x the parameter count, NTv2-500m features a *smaller* memory footprint with comparable FLoating-point OPerations (FLOPs) to Caduceus PS. NTv2-500m sits at 12.58 TFLOPs/example compared to Caduceus-PS at 7.84 TFLOPs/example. Borzoi-esque architectures like NTv3-650m sit between the two.

In summary, compute spent increasing context size is a tradeoff with parameter count or hyperparameter exploration.

### Results on new tasks in GUANinE v1.1

Our results in Table 3 suggest that while gLMs excel at region-level conservation estimates, their dominance on variant-level tasks is less clearcut. While notably, none of Enformer, Borzoi, or Sei approach the performance of gLMs like NTv2-500m on cadd-snv deleteriousness prediction, the performance of both GPN-MSA and phyloP-30way confirms that more information can be extracted from multiple sequence alignments (MSAs) than is captured by pure gLMs. The sequence-only follow-up to GPN-MSA, PhyloGPN, only partially addresses this gap.

Turning to the ‘low’ performance of CADD v1.7 on cadd-snv, relative to two of its inputs, phyloP-30way and -100way, we note that per the CADD methodology (Nazaretyan *et al.* 2025), their best-performing models on external validation data are only partially fit to the deleteriousness task. Specifically, CADD 1.7 undergoes 13 iterations of L-BFGS and is stopped early to *prevent* convergence. In varCADD, this early stopping is even more pronounced, with its authors performing only ∼3 iterations of L-BFGS across training sets.

We suggest this intentional *under*fitting of CADD as the reason it outperforms conservation-based approaches on our variant pathogenicity task, clinvar-snv. We suspect the failure of other models to generalize from deleteriousness to pathogenicity is due both to intrinsic differences between the tasks and to phenomena like the construct validity of the ClinVar database for pathogenicity (Ho and MacRae 2009). In fact, while we do not report scores, we found that cadd-snv-optimized models show little to no predictive power on clinvar-snv, a finding we did not originally anticipate.

### Evolutionary distal sequences can improve gLM performance

Next, we consider the matter of pretraining corpus, which is known to substantially impact the quality of the downstream model (Kaplan *et al.* 2020). A reasonable question is whether training on non-human sequences can improve performance on human tasks. We provide several points of evidence to suggest that yes, even distally related genomes, in sufficient quantity, can improve downstream model performance.

The most direct evidence comes from contrasting DNABERT-2 and NTv2-100M, seen in Table 2. Given their primary difference is the latter’s broader pretraining corpus, we attribute NTv2-100M’s success over DNABERT-2 to the gradients extracted from distally related, non-human genomes. As a corollary, we observe that GENA-LM-bert-base-t2t-multi does not exhibit catastrophic forgetting on GUANinE tasks, despite its fine-tuning on a combination of non-human genomes.

We additionally observe that gLMs pretrained on small genomes appear to underperform those pretrained on unrelated, yet larger genomes. Specifically, GENA-LM-bert-base pretrained on hg38 outperforms GENA-LM-bert-base-yeast, trained only on yeast, even on the gpra-c and -d tasks in yeast (Table S5). Pretraining on a large collection of fungal genomes may be optimal, but corpus size appears to matter at least as much as genome relatedness when pretraining gLMs.

### Digging into Evo2

Despite its superior performance at high-context sizes relative to other gLMs, Evo2_7B is one of the more difficult models to use. To assist other modelers, we detail a number of insights based on its performance on a large-scale benchmark like GUANinE.

Specifically, we found that Layer 26 of Evo2_7b performed the best across tasks. Embeddings extracted from layers 25 and 27 *rarely* exhibited better performance on the development set, but we observed this was due to overfitting, rather than truly better performance. Strikingly, we observed a *stark* decrease in performance when using embeddings from deeper layers. We believe this may be due to particular issues in Evo2’s architecture, be it missing residual connections or specialized layers. We did not observe this degradation in any other models, including the Mamba-based Caduceus models. We inferred single examples on 2× L40 GPUs (engineering choices prevent Evo2 models from being inferenced on GPUs released by Nvidia before 2022.) at 32 kbp of input size by **deleting** deeper layers. We did not attempt the maximum 1 Mbp input size due to computational infeasibility—in natural language processing, successful 7B parameter architectures like Mistral (https://mistral.ai/news/announcing-mistral-7b) target more modest context sizes.

### Digging into Sei

Several factors drive Sei’s strong performance despite its small context size, including its high parameter count (approaching 900M) and immense supervision (21 907 output tracks). Additionally, Sei is lightweight despite its high parameter count: it requires 105.94 GFLOPs/example compared to Enformer’s single-bin inference of 31.16 TFLOPs/example (Enformer parallelizes over genomic windows for *training* efficiency; inferring three consecutive bins is a slight increase to 32.2 TFLOPS/ex.—a trend of ≈ .02 TFLOPS per additional bin). Sei is at a disadvantage when measuring FLOPs per bp, since it measures only 26.49 MFLOPS/bp compared to Enformer’s 158.5.

However, this brings us to a key advantage of Sei: its model complexity. In fact, it tallies 222.5 kiloparameters/bp compared to Enformer’s 1.28, as listed in Table S5. We attribute Sei’s success to this increased model complexity, despite lower input size and operational intensity.

### Digging into NTv3

Remarkably, the NTv3-pre series underperforms *other language models*—including its precedent NTv2s—on GUANinE. This is most notable on cons30 and cons100 in Table 2.

However, GUANinE v1.1 shows that *after* additional supervision (NTv3-100m-post and NTv3-650m-post), even the smaller, lower-context NTv3-100M-post model outperforms both Enformer and Borzoi on functional tasks like dnase-prop and ccre-prop  *and* offers gains on variant deleteriousness predictions in cadd-snv. This is strong evidence that gLM pretraining benefits downstream models, even if the architecture underperformed on language modeling. At lower FLOPs, 12× the number of organisms, and the choice between the lightweight 100M and performant 650M models, NTv3 represents a promising approach to combining self-supervised pre-training with supervised post-training.

### Initial results with AlphaGenome

At time of evaluation, AlphaGenome had not been fully released, and its API expressly prohibited large-scale (N>1M) evaluations. As such, we have only evaluated AlphaGenome on clinvar-snv, despite our eagerness to evaluate it across all tasks. On clinvar-snv, we find that the AlphaGenome *framework*, that is, its unique approach to pre- and post-processing variant scoring, improves performance even for other models.

In particular, we note the clinvar-snv score of additively combining Pangolin and Sei-Aggr, the latter simplified to the zero-shot regime through the same variant scoring approach as AlphaGenome (Avsec *et al.* 2026). The two together see 10 kbp of context, but outperform AlphaGenome’s 1 Mbp of context on clinvar-snv. This finding is in line with previous reports that large-context models do not fully make use of distal sequence context (Karollus *et al.* 2023), meaning that small-context models often match their performance. Additionally, our results suggest that at least on pathogenicity prediction, the benefit of AlphaGenome’s *model* may come largely from its marriage of splicing tasks with functional data.

We anticipate AlphaGenome’s scorer will be widely applied to other models and other tasks, and we would recommend further research into similar structured dimensionality reductions.

## Discussion and conclusion

No one genomic model can accomplish every task, and our results highlight the need for benchmarks like GUANinE, unhindered by sample size or reporting bias during model publication, to thoroughly evaluate pretrained models.

Controlling for other variables, context size should improve model performance; however, we note that models like Evo2_7B may achieve a Pyrrhic victory in this regard. Research groups attempting to use such a model may be restricted to small sample sizes or a shallow analyses, given the immense computation per single inference. Similarly, AlphaGenome could reasonably infer a few thousand variant effects for a clinical geneticist, but neither is practical for a computational researcher experimenting on model behavior through millions of inferences. For that, we would point to NTv2, NTv3, or Sei instead.

We would also suggest that this limitation of high-context models during inference may be a limitation during training. On GUANinE, the supervised Sei outperforms Enformer at functional element annotation, while the unsupervised NTv2 outperforms NTv3-pre on estimating sequence conservation. Choices like parameter count, pretraining corpus, or architecture seem to matter more than context size across our evaluations.

Given the complementary strengths of supervised models and gLMs, we suggest combining the two going forward. In natural language processing, the predominant paradigm is the self-supervised pretraining of a large model, which can then be fine-tuned (or ‘post-trained’) on downstream tasks. This was investigated in GUANinE v1.0 with fine-tuned hgT5 language models (Robson and Ioannidis 2023), but it was not until NTv3 that supervised-only genomic models were superseded, at least on many of the tasks evaluated here. In fact, both GENA-LM-bert-base-t2t-enformer and NTv3-650M-post exceed their original language models when annotating functional elements and modeling sequence conservation, although they appear to gain genome specialization (neither was *supervised* on fungi) given their decrease in performance on gpra-c and -d.

Additionally, it remains to be seen how embeddings of more specialized models, such as those dedicated to promoter and UTR elements (Shearer *et al.* 2025), those restricted to mRNA (Agarwal and Kelley 2022), or those dedicated to single-cell data (Lal *et al.* 2025) would fare on general noncoding benchmarks like GUANinE. We would expect that specialization reduces training time at the cost of downstream flexibility.

In conclusion, the v1.1 expansion of the GUANinE genomic sequence-to-function benchmark has illuminated dozens of novel insights for the field of genome sequence modeling. Chief among them is clarity regarding the roles of supervised sequence-to-function and unsupervised genomic language models: the two have distinct strengths and use cases, and since both allow for unique perspectives on variant effects, we believe the two can be intertwined to enable models to more fully comprehend a genomic sequence or the effects of variation.

## Supplementary Material

btag427_Supplementary_Data

## Data Availability

The GUANinE benchmark can be accessed at https://huggingface.co/guanine. User guides and task details can be found at https://guanine.readthedocs.io/en/latest/.
