# Supplementary material for: GUANinE v1.1 reveals complementarity of supervised and genomic language models
Source: Bioinformatics. 2026 Aug 21;42(Suppl 2):btag427. doi: 10.1093/bioinformatics/btag427 (PMC13501288; doi:10.1093/bioinformatics/btag427)
Supplement: Supplementary file 1 [file btag427_supplementary_data.pdf]

Supplementary Models Information

We once again enumerate newly evaluated models, this time complete with citations.

1. Two ChromBPNet models, Fold 0 and Fold 3, trained on bp-resolution ATAC-seq data from the GM12878 cell lines from Pampari et al. (2025) We also include the ChromBPNet training track ENCFF180XQC from Pampari et al. (2025) as a baseline.
2. DNABERT-2 genomic language model (gLM) from Zhou et al. (2023)
3. PhyloGPN phylogenetic-loss gLM from Albors et al. (2025)
4. All four Nucleotide Transformer v2 (NTv2) gLMs from Dalla-Torre et al. (2025)
5. Three Nucleotide Transformer v3 (NTv3) gLMs and two supervised derivatives from Boshar et al. (2025)
6. The ensemble-distilled Enigma model from Jung et al. (2025)
7. Six of the GENA-LM family of gLMs, plus one version fine-tuned on Enformer's training data, from Fishman et al. (2025)
8. The contrastive dna2vec of 'Embed-Search-Align' from Holur et al. (2025)
9. The supervised CNN Sei from Chen et al. (2022)
10. Two Mistral-DNA and two ModernBERT models from Mourad and Batut
11. Two of the Caduceus genomic language models, PS and PH, from Schiff et al. (2024)
12. Two of the Evo2 gLMs, including a second evaluation of Evo2_7b at a higher-context size, from Brixi et al. (2025)
13. Two of the gLMs from the AIDO group, as presented by Ellington et al. (2025)

Additionally, as part of the newly released `cadd-snv` and `clinvar-snv` tasks, we include a handful of additional models, such as:

1. CADD v1.7, an annotation-supervised deleteriousness model trained with simulation-augmented data from Schubach et al. (2024)
2. Pangolin, an RNA-splicing model developed by Zeng and Li (2022)
3. GPN-MSA, a conservation-centric MSA-to-function model from Benegas et al. (2025)
4. AlphaGenome, a supervised variant interpretation framework by Avsec et al. (2025)
5. Variant Effects Predictor (VEP) build 115, a baseline pipeline from McLaren et al. (2016)

Supplementary Tables

Table 5. Key:
B $\rightarrow$ non-neural Baseline | M $\rightarrow$ MSA-based method | S $\rightarrow$ Supervised pretraining | U $\rightarrow$ Unsupervised pretraining
F $\rightarrow$ Full fine-tuning | X $\rightarrow$ 1% evaluation | " " $\rightarrow$ zero-shot
$=$ $\rightarrow$ exactly | $\approx$ $\rightarrow$ rounded to | $\sim$ $\rightarrow$ on the order of
† Computationally infeasible
Omnibus results for the newly constructed tasks released in `GUANinE` v1.1. As discussed in the main text, that `clinvar-snv` has no training set, so if models are not zero-shot, they are calibrated to the development set as described in Methods. The best reported score, along with any close scores, are **bolded** per task. We underline the second best score(s).

Model	Reg.	Eval.	Context	kPar./bp	dnase-propensity ρ	ccre-propensity ρ	ccre-subtask dnase ρ	ccre-subtask ctcf ρ	ccre-subtask h3k27ac ρ	ccre-subtask h3k4me3 ρ	conservation 30 ρ	conservation 100 ρ	gpra-c ρ	gpra-d ρ
GC-content	B		511 ± 1	≈ 0.00	20.5533	12.9891	13.0236	12.6525	13.2115	13.0690	38.1307	39.5828	52.9688	56.9824
5-mer LinSVR	B	F	511 ± 1	≈ 0.00	36.3022	26.3708	23.3577	27.6775	27.7758	26.6722	49.3504	45.0548	69.4348	73.6469
5-mer *k*NN	B	F	511 ± 1	∞	31.9876	23.3579	22.0112	24.5605	23.8530	23.0069	52.5776	44.5351	58.5608	62.1082
★ T5		F	511 ± 1	1507	33.0548	27.4643	26.8736	28.2986	27.1943	27.4908	52.5576	44.0542	**84.6738**	**88.5912**
DeepSEA	S	X	$= 1$ k	52.8	34.6927	23.2158	22.7526	24.0649	23.3712	22.6745	49.6163	43.4004	68.6607	72.9729
Beluga	S	X	$= 2$ k	74.8	64.9577	49.6688	44.5226	51.6006	52.5047	50.0473	58.6143	48.8816	66.5479	69.9363
Basenji2	S	X	≈ 55 k	0.55	60.5149	56.4568	56.4814	56.3126	57.3374	56.3651	71.0417	59.1229	69.5484	72.9872
Enformer	S	X	≈ 197 k	1.25	64.9164	55.3816	53.0151	55.9015	54.1682	58.4416	74.4358	62.2344	70.6259	74.1311
Enformer (Pre-output)	S	X	≈ 197 k	1.17	68.0818	55.0775	52.4223	54.6072	54.9317	58.3487	74.6815	62.4178	70.6976	74.2039
Borzoi	S	X	≈ 524 k	0.35	70.6892	57.6904	55.2756	<u>58.5863</u>	56.6941	<u>60.2057</u>	71.5495	59.6878	71.5318	75.1785
ENCFF180XQC	B		511 ± 1	0	24.1638	34.8485	33.3688	35.2081	34.4924	36.3248	4.0874	6.4552	–	–
ChromBPNet-ATAC-F0	S	X	= 2114	3.00	41.4931	40.4249	39.918	40.6751	40.4186	40.6878	47.7772	41.4890	71.0865	74.9317
ChromBPNet-ATAC-F3	S	X	= 2114	3.00	40.5515	40.4745	40.3492	41.1234	40.0861	40.3394	47.4057	40.3217	70.6157	74.2397
Sei	S	X	$= 4$ k	222	70.4688	56.3547	53.9095	57.2327	55.2832	58.9933	63.1947	51.5780	68.8511	72.1761
Enigma-Distilled	S	X	≈ 524 k		65.7599	51.7599	49.8365	51.9146	50.9937	54.2947	63.0186	45.8052	67.9518	71.4524
DNABERT-2	U	X	~ 12 k	~ 9.7	41.6618	32.7470	31.9049	33.3137	32.0785	33.6911	60.5895	50.3579	69.8810	73.9089
PhyloGPN	U	X	= 481	173	40.4750	18.4041	17.2970	19.7719	17.8236	18.7240	53.2182	43.1253	66.8694	70.7405
NTv2-50m	U	X	~ 12 k	~ 4.2	43.6408	37.4346	36.0646	37.8445	37.5032	38.3260	65.8741	55.1823	71.4157	75.1369
NTv2-100m	U	X	~ 12 k	~ 8.3	42.0778	35.7199	33.5139	36.7799	35.5785	37.0074	69.2337	57.9283	71.3679	75.1096
NTv2-250m	U	X	~ 12 k	~ 20.8	43.6973	36.0699	34.3708	36.5686	35.7485	37.5915	73.2828	61.0874	72.4360	76.2963
NTv2-500m	U	X	~ 12 k	~ 41.7	46.6071	38.1982	36.6067	38.2818	37.7142	40.1903	<u>75.5842</u>	<u>63.5168</u>	72.6726	76.5765
[0.1pt] NTv3-8m-pre	U	X	≈ 8.2 k	0.98	40.3151	32.8373	31.3699	33.3542	32.2069	34.4184	60.1950	48.6597	71.7001	75.3956
NTv3-100m-pre	U	X	≈ 8.2 k	12.2	42.6554	33.8883	31.5465	34.5960	33.6909	35.7197	64.2313	51.4815	72.9524	76.7832
NTv3-650m-pre	U	X	≈ 8.2 k	79.3	45.2498	34.8631	32.9167	35.3547	34.8019	36.3790	69.6601	56.6031	74.7650	78.4196
NTv3-8m-pre	U	X	≈ 32.8 k	0.24	40.3548	33.8244	32.0331	34.4430	33.4083	35.4130	61.3461	49.3171	71.6239	75.3517
NTv3-100m-pre	U	X	≈ 32.8 k	3.05	42.7267	34.6183	32.5645	35.0503	34.6352	36.2231	65.7691	52.9409	73.0106	76.8087
NTv3-650m-pre	U	X	≈ 32.8 k	19.8	45.5815	35.8263	33.7283	36.5234	35.4747	37.5787	71.4900	58.5516	74.8158	78.4714
NTv3-100m-post	U+S	X	≈ 32.8 k	3.66	<u>72.4960</u>	<u>59.3062</u>	<u>57.2026</u>	**59.9494**	<u>58.2672</u>	<u>61.8056</u>	72.0146	59.6267	69.9982	73.5891
NTv3-650m-post	U+S	X	≈ 32.8 k	20.7	**75.2947**	**59.9739**	**58.4435**	**59.9849**	**59.3887**	**62.0788**	73.2703	60.5921	71.7652	75.4116
[0.1pt] GENA-LM-bert-base	U	X	~ 4.5 k	~ 24.4	43.2094	36.5813	35.2246	36.8412	36.0472	37.2644	65.3625	55.0575	70.2835	74.0042
GENA-LM-bert-base-yeast	U	X	~ 4.5 k	~ 24.4	32.3436	28.2020	27.8107	28.5989	28.0560	28.3426	51.6697	43.9358	63.9957	68.0473
GENA-LM-bert-base-t2t	U	X	~ 4.5 k	~ 24.4	42.8185	34.8672	34.5074	34.2673	34.4766	36.2176	66.6483	55.9618	71.0697	74.8112
GENA-LM-bert-base-t2t-enf	U+S	X	~ 4.5 k	~ 24.4	54.2948	48.7415	47.5286	48.6994	48.5088	50.2292	68.1750	56.5446	70.6333	74.2133
GENA-LM-bert-base-t2t-multi	U	X	~ 4.5 k	~ 24.4	42.8573	34.7512	34.1537	34.6447	34.1553	36.0509	65.8067	55.5404	71.2714	74.9640
GENA-LM-bert-large-t2t	U	X	~ 4.5 k	~ 74.7	44.4264	36.1742	35.3316	35.1839	36.1485	38.0329	71.6328	59.8846	71.5104	75.2759
GENA-LM-bigbird-base-t2t	U	X	~ 36 k	~ 3.05	44.1009	37.3342	35.8006	36.8634	37.5124	39.1605	73.6004	61.5070	71.3673	75.0451
[0.1pt] ESA-dna2vec	U	X	= 500	108	26.0047	17.4341	16.0428	18.8609	16.8940	17.9387	44.0860	39.7871	59.1203	62.7899
[0.1pt] Mistral-DNA-17m-hg38	U	X	~ 10 k	~ 1.7	34.5188	31.9774	30.8939	32.6777	31.1073	33.2308	53.7361	45.7752	67.1016	70.8973
Mistral-DNA-422m-hg38	U	X	~ 10 k	~ 42.2	39.2215	36.0627	35.3610	35.7797	35.7223	37.3879	60.5367	50.7310	69.4335	73.1728
Modern-BERT-37m-hg38	U	X	~ 10 k	~ 3.7	25.7812	20.0403	18.5395	21.4866	19.3491	20.7859	42.4283	39.2057	55.5611	59.5683
Modern-BERT-117m-hg38	U	X	~ 10 k	~ 11.7	35.3822	30.8061	31.0741	31.3223	30.2893	30.5387	53.5316	46.7186	66.5842	70.4314
[0.1pt] Caduceus-PS	U	X	≈ 131 k	0.06	42.9735	41.3231	39.7175	41.0856	41.7602	42.7293	74.3749	62.3797	72.4355	76.0289
Caduceus-PH	U	X	≈ 131 k	0.06	41.6935	39.1782	38.1810	39.5764	39.8438	39.1114	72.5137	60.9183	72.1815	75.9232
[0.1pt] Evo2_1B_base	U	X	≈ 8.2 k	122	41.7896	40.2332	39.0054	40.8129	39.2768	41.8379	68.4935	55.2246	72.6487	76.3823
Evo2_7B_base	U	X	≈ 8.2 k	854	46.9047	45.5150	44.4768	46.2969	44.7764	46.5100	74.0702	61.8923	<u>75.5008</u>	<u>79.0651</u>
Evo2_7B	U	X	≈ 32.8 k	214	47.1553	47.0887	45.7063	47.3957	46.965	48.2879	**76.7131**	**65.3087**	†	†
[0.1pt] AIDO.DNA_300m	U	X	$= 4$ k	75	42.6601	35.7702	35.3850	35.2563	35.6643	36.7754	60.9914	51.5037	73.5186	77.2525
AIDO.DNA_7b	U	X	$= 4$ k	1750	42.7731	36.8271	36.8119	36.4697	36.4854	37.5414	59.1557	49.5802	69.3635	73.0252

Model	Reg.	Eval.	Context	**cadd-snv** ρ
GC-content	B		511 ± 1	1.4665
Naïve VEP 115 Conseq.	B		–	0.1565
deltaSVM GM12878 DHS	B		19	7.6083
GPN-MSA	M			**29.1221**
GPN-MSA (Position)	M			26.6472
phyloP-100way	M			23.2761
phyloP-30way	M			<u>28.7228</u>
CADD v1.7	S		–	18.1545
CADD v1.7 (Position)	S		–	19.6639
ChromBPNet-ATAC-F0	S	X	2114	17.2853
ChromBPNet-ATAC-F3	S	X	2114	16.7586
Enformer	S	X	≈ 197 k	17.0984
Borzoi	S	X	≈ 524 k	20.9877
Sei	S	X	$= 4$ k	19.9110
Evo2_1B_base	U	X	≈ 8.2 k	24.5028
NTv2-50m	U	X	~ 12 k	21.8891
NTv2-100m	U	X	~ 12 k	23.8862
NTv2-250m	U	X	~ 12 k	25.4037
NTv2-500m	U	X	~ 12 k	26.4742
NTv3-8m-pre	U	X	≈ 8.2k	18.4170
NTv3-100m-pre	U	X	≈ 8.2k	22.8381
NTv3-8m-pre	U	X	≈ 32.8k	19.5227
NTv3-100m-pre	U	X	≈ 32.8k	22.8564
NTv3-100m-post	U	X	≈ 32.8k	22.1362
PhyloGPN	U		$= 481$	9.8049
PhyloGPN	U	X	$= 481$	23.5960

Table 6. Proxy deleteriousness is dominated by conservation scores, in particular the neural GPN-MSA and phyloP-30way. Those same conservation scores underperform on variants in `clinvar-snv` – showcasing the strategic *underfitting* to deleteriousness the CADD developers rely on for pathogenicity performance.

Model	Reg.	Context	**clinvar-snv** ρ
GC-content	B	511 ± 1	11.5278
Naïve Conseq. Type	B	–	76.7449
phyloP-30way	M		66.0851
GPN-MSA	M		80.7211
phyloP-100way	M		81.5065
PhyloGPN	U		67.5918
AlphaGenome-K562	S	≈ 1.05 M	<u>93.1695</u>
CADD v1.7 (Position)	S	–	83.6767
CADD v1.7	S	–	84.6379
Pangolin	S	≈ 10 k	85.6332
Sei-Aggr.	S	$= 4$k	50.8500
Sei-Aggr. + Pangolin	S+S	≈ 10 k	**95.1299**

Table 7. Due to its splicing-biased provenance, `clinvar-snv` scores are dominated by splicing effects and, due to small sample size, low-dimensional reductions like AlphaGenome's variant scorer. This same scoring strategy, when combined with Sei in 'Sei-Aggr.' and the splicing model Pangolin, outperforms even AlphaGenome.

Supplemental Methods

Model parameter counts were validated manually in PyTorch. Flop calculation estimates were provided by Calflops (xiaoju ye, 2023). Note that while a decent indicator of model compute, flops are an imperfect metric and definitions vary from machine to machine.

Descriptions of Evaluated models

ChromBPNet models

Pampari et al. (2025) introduced compact, cell-line-specific supervised models tailored to *decision explainability*, i.e. identifying putatively causal motifs. The ChromBPnet family of models are bp-resolution CNNs that accept 2,114 bp of input and output a factorized 1,000 bp of signal. We choose to include two folds of the ATAC-seq trained models based on the GM12878 cell line, which has a high 572M read depth. Fold_0 is the preferred model in Pampari et al. (2025), although we also report the *unbiased* Fold_3, which is consistent with GUANinE's train-test split. As the scalar output is less transferable to tasks in GUANinE v1.0, we pruned layers after add_7 and used the ultimate 512-dimensional embeddings for ChromBPNet models. This leaves a total of ≈ 6.34M parameters, much smaller than other models.

ENCFF180XQC baseline

In addition to the ChromBPNet models, we evaluate the pre-processed signal input to the models, namely, ENCODE track ENCFF180XQC. We report this baseline to give readers insight into the ATAC-seq chromatin accessibility signals underpinning GUANinE tasks (i.e. construct validity), as well as to provide a new form of baseline – raw experimental data.

DNABERT-2

A followup to the early gLM DNABERT, the second version maintains a comparable performance footprint but at higher context sizes through byte-pair encoding tokenization. The model is trained via unsupervised masked language modeling across 135 species spanning multiple phylogenetic kingdoms – without any apparent deduplication (the authors merely remove sequences with 'N'). DNABERT-2 follows the original BERT-base architecture but with minor tweaks, such as a position-aware attention mechanism. We aggregate per-bp final-layer embeddings of dimension 768, and the model consists of 117M parameters.

Nucleotide Transformers v2

Trained across a broad set of 850 species, the second iteration of the Nucleotide Transformer gLMs use k-mer tokens (predominantly 6-mers) on moderate context sizes with an enhanced BERT-style architecture. Trained via masked language modeling, the corpus of NT models is an augmented with observed variation, including individual haplotypes from 1000 Genomes (The 1000 Genomes Project Consortium, et al., 2015). Similar to DNABERT-2, there is otherwise minimal filtering of the training corpus. Model parameters in the v2 family are included in the name of each model, e.g. '100M,' and correspond to hidden dimensions of 512, 512, 768, and 1024, respectively. We abbreviate these models 'NTv2' throughout this work.

Nucleotide Transformers v3

Pretrained across the same corpus as Evo2, the third iteration of the Nucleotide Transformer gLMs use a U-net style architecture across large context sizes, not unlike a language model version of Borzoi. We abbreviate this family of models 'NTv3' throughout this work. After pretraining, these models are fine-tuned or "post-trained" by their creators to further specialize them into a set of 24 organisms. These 'NTv3-post' variants have a slightly higher parameters due to the output head, reaching 120M parameters for the NTv3-100M-post and 680M parameters for the NTv3-650M-post model.

Enigma

This large-context supervised model bridges Borzoi and AlphaGenome, while incorporating considerable architectural tweaks for computational efficiency. Similar to AlphaGenome (Avsec et al., 2025) it uses ensemble-distillation to boost performance and robustify results. Note that compared to Borzoi it has a slightly higher 202M parameter count, of which the final output layer is a modest 0.7% due to its compact embeddings and reduced output track count.

ESA-dna2vec

This contrastive model is distinct for its dedication to sequence embedding – and its usage of only $\approx$ 2% of hg38 during training (Holur et al., 2025). While it is relatively underpowered compared to other methods, it showcases the potential for contrastive pretraining in genome modeling. The model sits at a total of $\approx$ 54M parameters, of which the initial embedding layer constitutes 18.9%.

PhyloGPN

This MSA-inspired model is specialized to predict centered SNVs given 480 bp of surrounding context. While the training loss is weighted via MSA information, forward inference is performed on sequences alone, enabling its straightforward evaluation as a sequence model in GUANinE. The model totals just over 83 million parameters, of which the output logit layers make up $\approx$ 0.007%. Aside from the `clinvar-snv` task, which logits are ideal for, we use the 960-dimension mean-pooled embeddings. We report both embedding and zero-shot logit scores for `cadd-snv`.

GENA-LM

A family of models and architectures, the GENA-LM series uses byte-pair encoding across different genomes, including the human t2t genome (Nurk et al., 2022), during gLM pretraining. Pretraining genomes were typically augmented with SNVs from 1000 Genomes or similar. Architectures span BERT-base, BERT-large, and BigBird, which offers a much higher input context than traditional transformer encoders. We do not evaluate the sparse implementation of BigBird, but those willing to install it will likely see speedups. Of architectures evaluated, the models consist of 111M, 337M, and 113M parameters, respectively.

Caduceus

The two Caduceus gLMs evaluated, PH and PS, evaluate reverse-complement-augmented and RC-equivariant architectures, respectively, using versions of the long-range Mamba SSM architecture (Gu and Dao, 2024; Schiff et al., 2024). Both models are trained with only the hg38 reference

genome – a significant disadvantage compared to many other gLMs in `GUANinE` v1.1. Each model consists of ≈ 7.7M parameters, making them among of the smallest high-context models evaluated.

Evo2
A slow yet potent family of gLMs evaluated, the Evo2 models use a custom SSM-esque architecture to achieve high context size inference on a broad yet well-pruned set of genomes. We do not attempt to infer Evo2_7B beyond 32 kbp of input, nor do we evaluate Evo2_40B, as for a *single example*, either requires more than 192 GB of VRAM on Lovelace+ Nvidia GPUs (computational constraints limited us to 4x L40 GPUs). The 1b_base and 7B_base models operate on 8.2 kbp input sizes; we found that uncased (uppercase) sequences yielded much better performance despite the models having been trained on repeat-masked sequences.

Sei
The third iteration of DeepSEA, Sei continues the tradition of flattening a large convolutional network into an output layer – albeit with a more sophisticated non-linear output than previous versions. Uniquely, Sei also predicts 21,907 tracks, making it the most-supervised model evaluated in v1.1, although Borzoi or AlphaGenome have different types of data compared to Sei (e.g. RNA-seq or splicing, while Sei is primarily ChIP-seq). Being intensely concentrated on its 4 kbp input size, Sei consists of nearly 890M parameters, of which 91.7% are located in the quadratic output layers. Given this density of weights in the output layers, we use outputs, not embeddings, for all Sei results.

AIDO
As part of a larger AI Digital Organism ('AIDO') project for model generation, AIDO.DNA models are gLMs trained on nearly the same corpus as the Nucleotide Transformer models. Both the 300M and 7B formulations are trained on 4 kbp of input. In contrast to the reported results of Ellington et al. (2025), we do not fine-tune the full models, nor do we select the optimal checkpoint for each evaluation task. This may partially explain the near-equivalence of the two's performance in our results.

Mistral-DNA and ModernBERT
Pedagogically-inclined models developed by Raphael Mourad (Mourad and Batut), Mistral-DNA and ModernBERT use sophisticated architectures on minimally preprocessed hg38 datasets, but still achieve respectable scores on `GUANinE` tasks. We attribute this not solely to the power of modern LM architectures, but also to the underexplored hyperparameter space of gLMs more generally.

GPN-MSA
A multiple-sequence-alignment-to-function model, GPN-MSA is a sophisticated approach to sequence conservation modeling. Due to its not directly modeling unaligned sequences, we do not attempt to evaluate GPN-MSA on most tasks – instead, we use the precomputed genome-wide scores computed by Benegas et al. (2025). See `https://huggingface.co/datasets/songlab/gpn-msa-hg38-scores`.

Calibrating AlphaGenome and Sei-Aggr.

As described, but not detailed, in the AlphaGenome preprint (Avsec et al., 2025), we utilized a small-N 'calibration' of combined variant scorers derived by aggregating the output of models by experiment type. For Sei, we found that aggregating experiments with at least 20-50 examples, coupled with pruning the rarer experiments, produced the best results (using '20' as the threshold shrinks the dimensionality from 21,907 → 109).
For AlphaGenome, we selected all tracks for a single cell line for aggregation, the well-represented K562 line, per the guidance by the authors(Avsec et al., 2025).
Once a low-dimensional scorer is achieved, for either the K562-outputs of AlphaGenome or Sei, we finish calibration by using logistic regression fit to the development set to weight each experiment type into a final score, similar to Avsec et al. (2025).

Logarithmic feature intervals

As has been suggested by model developers (Avsec et al., 2021; Linder et al., 2025), inferencing with alternative-resolution-pooled features is essential for thorough model evaluation. For new models in `GUANinE` v1.1, we utilized a sequence of concentric, single bp-centered resolutions for embeddings or outputs (where possible, e.g. not Sei or variant approaches like AlphaGenome). These correspond to a sequence of $L(k) = 1 + 2^k$ bp, or a series of $[1, 3, 5, 9, 17, 33, 65, \cdots, \lceil \log_2 \text{Context Size} \rceil]$ regions, with the final being equivalent to mean-pooling.
Note that for variable token-length models like DNABERT-2 or the NTv2 family, we use partially-weighted tokens when region boundaries fall in the middle of a token (e.g. 1 bp of a 3bp token $= 1/3$).
For coarse-resolution models like Enformer or Borzoi, we report the best of 1 or 3 bins, etc, see previous work for details (robson and Ioannidis, 2025).

Notes on Spearman's ρ

We unconventionally choose to report Spearman's ρ for our Boolean classification tasks, `cadd-snv` and `clinvar-snv` . We justify this with both the ease of interpretability (as ρ^2 is the proportion of variance explained by ranks), and the fact that it reduces to the point-biserial correlation, a well-studied distribution for class-balanced situations such as ours (Tate, 1954). We point readers interested in the numerical of comparison point-biserial to other metrics to Salgado (2018). Additionally, we use continuous outputs for scoring, i.e. `predict_proba` for scikit-learn models like logistic regression.

Datasets

`clinvar-snv`
The `clinvar-snv` dataset was designed to be harmonious with existing pathogenicity and deleteriousness detection methods, rather than to be a (possibly ill-defined) objective measure of pathogenicity grading.
To that end, we began by cross-referencing the 2+ star non-coding variants used in CADD v1.7 (compiled June 2023, (Schubach et al., 2024)) with the more up-to-date set of ClinVar database variants from 24 August of 2025. Our test

set consists of only the pathogenic and benign variants present in both (i.e. still considered accurate), while our development set is are those added to ClinVar since June 2023 – and thus not originally evaluated in CADD v1.7. This guarantees that CADD v1.7, and models fairly compared on our test set, do not benefit from data leakage when reporting scores on our `clinvar-snv` task.

This yields an imbalanced set of 58899 dev benign, 4601 dev pathogenic, 28900 test benign, and 5982 test pathogenic 2+ star variants, which we sought to class-balance via downsampling. Given the extreme feature imbalances present in the ClinVar database (substitution rate, VEP label, chromosome), we implement propensity score matching (Rosenbaum and Rubin, 1983) across the aforementioned features to downsample an optimized, class-balanced subset of benign variants. Propensity models were an ensemble of seven randomly sampled, class-balanced logistic regressions. Subsets were then derived by matching propensity scores via PuLP (Mitchell et al., 2011) and set to the maximum possible balanced number of pathogenic variants per chromosome.

The final distribution of splicing and intronic variants by pathogenicity label is illustrated by Supplementary Figure 3.

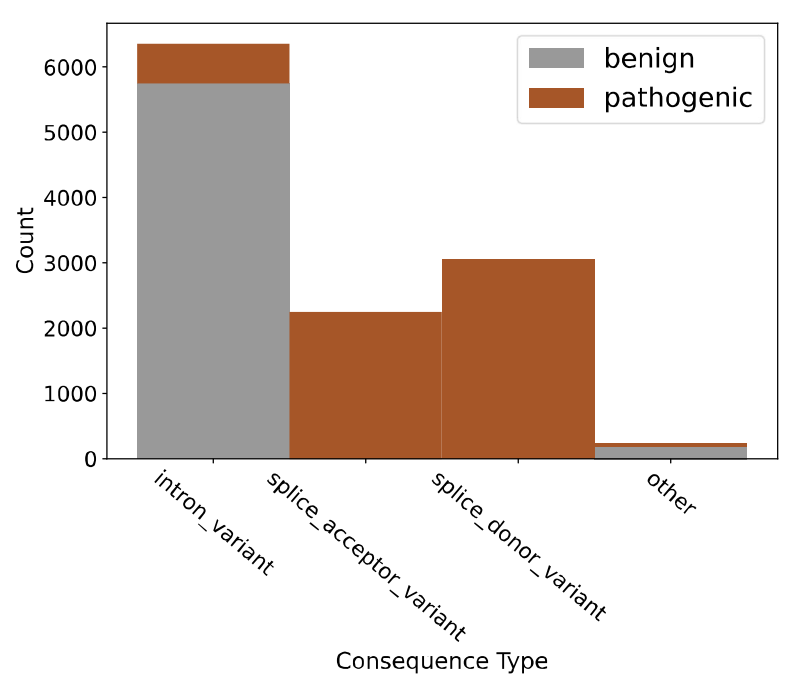

Figure 3 Variant consequence imbalances in `clinvar-snv` . These imbalances are representative of the ClinVar database and make the new `clinvar-snv` task most relevant for evaluating predictions of variant impact on splicing.

cadd-snv

We begin with the substitution-rate-balanced `hfs` set of varCADD (Nazaretyan et al., 2025), available in the varCADD Zenodo repository (link). This corresponds to a 50/50 split of proxy benign SNVs and proxy deleterious singletons (`s`), the former being a combination of evolutionary-derived variants (`h`) and frequent variants (`h`).

Four post-processing filters were then applied:

1. Removing coding variants (located in 'CDS' regions per GENCODE v48 (Mudge et al., 2024))
2. Deleting mislabeled *variants* according to the ClinVar database (e.g. removing CADD's proxy benign variants listed in ClinVar's 'Pathogenic' set)
3. Pruning leaked *positions* in the ClinVar database (e.g. coordinates present in the proxy benign set with a benign ClinVar alt-allele)
4. Downsampling proxy benign and proxy deleterious variants to be balanced per chromosome-substitution (e.g. 'Chr 22 G>A').

These specific filters imply a number of unique properties about the resulting data, most notably the inclusion of splicing variants and those located in UTR regions. Additionally, the orthogonalization of the ClinVar database and `cadd-snv` variants ensures that models trained or validated using `cadd-snv` have reduced risk of overfitting to `clinvar-snv` . We finally note that our chromosome-substitution balancing is more rigorous than the original varCADD procedure, although this represents an additional $\approx 2\%$ downsampling for `cadd-snv`. While a larger yet still balanced dataset could be achieved via filtering the `hfs-all` dataset, we opine that `GUANinE` is meant to be a benchmark, not a comprehensive training database. We would encourage model developers seeking to maximize performance to assemble the highest quality training data, then screen it against `GUANinE`'s `dev` and `test` splits for comparison. Dataset reductions, in order of derived, frequent, and (deleterious) singleton sets, are roughly:

1. Prune coding variants: $\approx -0.6\%, -0.8\%, -1.2\%$
2. Filter against any-star variant in ClinVar: -95, -108, -2646 (all less than 0.01%)
3. Downsampling to rebalance: $\approx -2.2\%, -2.2\%, -1.8\%$

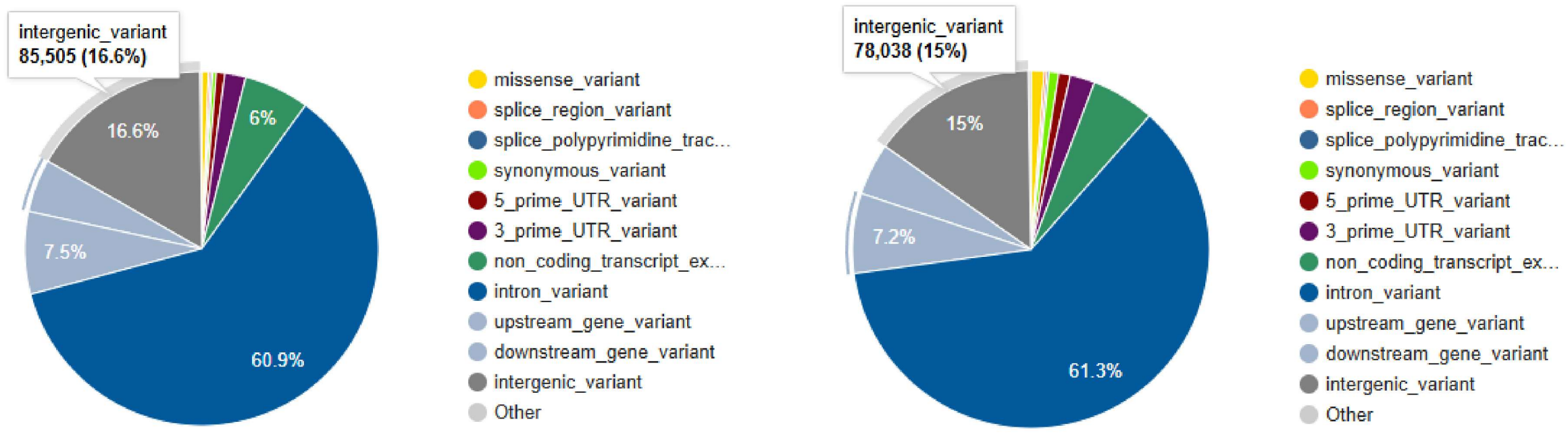

Figure 4 `cadd-snv` features dramatically more intergenic and non-splicing noncoding variants compared to `clinvar-snv` , which allows it to more broadly evaluate variant effect predictions. (a) Negative (proxy benign) `cadd-snv` variant consequence distribution, as predicted by VEP 115. (b) Positive (proxy deleterious) `cadd-snv` variant consequence distribution, as predicted by VEP 115. As seen by comparing (a) and (b), the task features a relatively minor consequence imbalance, which may increase its difficulty.

Few-shot `cadd-snv`

As in the original `GUANinE` benchmarks, we derive a 'few-shot' subset of the training data which corresponds to $\approx 1\%$ of the original training size (Zhang et al., 2016; robson and Ioannidis, 2023). To do this, we use perform stratified sampling proportionately on chromosome-substitution combinations to preserve, across features, the class balance of proxy benign vs proxy deleterious. In terms of bias-variance tradeoff, this reduces the variability of models fit to the few-shot subset, which should more accurately resemble said model's performance on the larger training set. Modelers should note this further entrenches the *per-observation* biases of the `cadd-snv` dataset[4] – those who are seeking to predict deleteriousness across *samples* would benefit from alternative sampling procedures (i.e. different frequencies of sampled variants).

Supplementary Note

Here we detail summaries of the six different tasks from `GUANinE` v1.0, grouped by type.

1. Regulatory elements: `dnase-prop` asks a model to assess tissue-agnostic DNase-hypersensitivity, corresponding to ENCODE v3's definition of a DNase-hypersensitive site (The ENCODE Project Consortium, 2020). Input sequences are taken from hg38, with the output being an ordinal 0-4 scalar of consensus-peak accessibility reflecting its degree of specificity across many cell lines. Its corollary, `ccre-prop` instead requires estimating the *functional properties* of those accessible sequences, such as candidate cis-regulatory element (cCRE) histone modifications like H3K4me3 (The ENCODE Project Consortium et al., 2020; The ENCODE Project Consortium, 2020). Many supervised models, from DeepSEA to Enformer, have seen the training and even testing data for these two tasks, making them in-distribution (robson and Ioannidis, 2025).

2. Conservation: `cons30` and `-100` require estimating the degree of conservation, or tendency to stasis, of an input hg38 sequence using an aggregated score of the popular phyloP metric (Pollard et al., 2010). The `30` consists of conservation defined across a 30-way mammalian multiple sequence alignment, primarily including primates, while the `100` consists of a 100-way alignment of vertebrate sequences, primarily mammals.

3. Synthetic promoters: The two synthetic tasks, `gpra-c` (complex) and `-d` (defined) correspond to two sets of large scale dual-reporter assay experiments measuring *gene expression* (Vaishnav et al., 2022; de Boer et al., 2020). Randomized promoters are prepended to reporter genes before being transformed into yeast.

Continuing in the direction of benchmark longevity, most tasks in `GUANinE` do not have publicly accessible test *labels*, given the risks of (inadvertently) overfitting to test sets, as well-documented in Kaggle contests (Blum and Hardt, 2015).

Previously reported model performances on `GUANinE` include the following (see the listed manuscripts for model details and evaluations):

1. GC-content, 5-mer Linear SVR, 5-mer kNN non-neural baselines (robson and Ioannidis, 2023)
2. T5 and hgT5 neural baselines (robson and Ioannidis, 2023)
3. DeepSEA, Beluga, and Basenji2 supervised CNNs (robson and Ioannidis, 2023)
4. Enformer and Borzoi supervised hybrid-convolutional models (robson and Ioannidis, 2025)

Note that as `cadd-snv` and `clinvar-snv` have not previously been described, we novelly report results on those tasks for several of these models.

Supplementary References

C. Albors, J. C. Li, G. Benegas, et al. A phylogenetic approach to genomic language modeling. In *Research in Computational Molecular Biology: 29th International Conference, RECOMB 2025, Seoul, South Korea, April 26–29, 2025, Proceedings*, page 99–117, Berlin, Heidelberg, 2025. Springer-Verlag. ISBN 978-3-031-90251-2. doi:10.1007/978-3-031-90252-9_7. URL `https://doi.org/10.1007/978-3-031-90252-9_7`.

Ž. Avsec, V. Agarwal, D. Visentin, et al. Effective gene expression prediction from sequence by integrating long-range interactions. *Nature Methods*, 18(10):1196–1203, Oct 2021. ISSN 1548-7105. doi:10.1038/s41592-021-01252-x. URL `https://doi.org/10.1038/s41592-021-01252-x`.

Ž. Avsec, N. Latysheva, J. Cheng, et al. Alphagenome: advancing regulatory variant effect prediction with

[4] Deleteriousness is not evenly distributed across substitutions, which themselves are variable across genomes.

a unified dna sequence model. *bioRxiv*, 2025. doi:10.1101/2025.06.25.661532. URL https://www.biorxiv.org/content/early/2025/07/11/2025.06.25.661532.

G. Benegas, C. Albors, A. J. Aw, et al. A dna language model based on multispecies alignment predicts the effects of genome-wide variants. *Nature Biotechnology*, Jan 2025. ISSN 1546-1696. doi:10.1038/s41587-024-02511-w. URL https://doi.org/10.1038/s41587-024-02511-w.

A. Blum and M. Hardt. The ladder: A reliable leaderboard for machine learning competitions. In F. Bach and D. Blei, editors, *Proceedings of the 32nd International Conference on Machine Learning*, volume 37 of *Proceedings of Machine Learning Research*, pages 1006–1014, Lille, France, 07–09 Jul 2015. PMLR. URL https://proceedings.mlr.press/v37/blum15.html.

S. Boshar, B. Evans, Z. Tang, et al. A foundational model for joint sequence-function multi-species modeling at scale for long-range genomic prediction. *bioRxiv*, 2025. doi:10.64898/2025.12.22.695963. URL https://www.biorxiv.org/content/early/2025/12/25/2025.12.22.695963.

G. Brixi, M. G. Durrant, J. Ku, et al. Genome modeling and design across all domains of life with evo 2. *bioRxiv*, 2025. doi:10.1101/2025.02.18.638918. URL https://www.biorxiv.org/content/early/2025/02/21/2025.02.18.638918.

K. M. Chen, A. K. Wong, O. G. Troyanskaya, and J. Zhou. A sequence-based global map of regulatory activity for deciphering human genetics. *Nature Genetics*, 54(7):940–949, Jul 2022. ISSN 1546-1718. doi:10.1038/s41588-022-01102-2. URL https://doi.org/10.1038/s41588-022-01102-2.

H. Dalla-Torre, L. Gonzalez, J. Mendoza-Revilla, et al. Nucleotide transformer: building and evaluating robust foundation models for human genomics. *Nature Methods*, 22(2):287–297, Feb 2025. ISSN 1548-7105. doi:10.1038/s41592-024-02523-z. URL https://doi.org/10.1038/s41592-024-02523-z.

C. G. de Boer, E. D. Vaishnav, R. Sadeh, et al. Deciphering eukaryotic gene-regulatory logic with 100 million random promoters. *Nat Biotechnol*, 38(1):56–65, Jan 2020.

C. N. Ellington, N. Sun, N. Ho, et al. Accurate and general dna representations emerge from genome foundation models at scale. *bioRxiv*, 2025. doi:10.1101/2024.12.01.625444. URL https://www.biorxiv.org/content/early/2025/01/11/2024.12.01.625444.

V. Fishman, Y. Kuratov, A. Shmelev, et al. Gena-lm: a family of open-source foundational dna language models for long sequences. *Nucleic Acids Research*, 53(2):gkae1310, 01 2025. ISSN 1362-4962. doi:10.1093/nar/gkae1310. URL https://doi.org/10.1093/nar/gkae1310.

A. Gu and T. Dao. Mamba: Linear-time sequence modeling with selective state spaces. In *First Conference on Language Modeling*, 2024. URL https://openreview.net/forum?id=tEYskw1VY2.

P. Holur, K. C. Enevoldsen, S. Rajesh, et al. Embed-search-align: Dna sequence alignment using transformer models. *Bioinformatics*, 41(3):btaf041, 02 2025. ISSN 1367-4811. doi:10.1093/bioinformatics/btaf041. URL https://doi.org/10.1093/bioinformatics/btaf041.

A. J. Jung, H. Zhu, R. Li, et al. Enigma: An efficient model for deciphering regulatory genomics. *bioRxiv*, 2025. doi:10.64898/2025.12.18.694875. URL https://www.biorxiv.org/content/early/2025/12/19/2025.12.18.694875.

J. Linder, D. Srivastava, H. Yuan, et al. Predicting rna-seq coverage from dna sequence as a unifying model of gene regulation. *Nature Genetics*, Jan 2025. ISSN 1546-1718. doi:10.1038/s41588-024-02053-6. URL https://doi.org/10.1038/s41588-024-02053-6.

W. McLaren, L. Gil, S. E. Hunt, et al. The ensembl variant effect predictor. *Genome Biology*, 17(1):122, Jun 2016. ISSN 1474-760X. doi:10.1186/s13059-016-0974-4. URL https://doi.org/10.1186/s13059-016-0974-4.

S. Mitchell, M. O'Sullivan, and I. Dunning. Pulp : A linear programming toolkit for python. *Optimization Online*, 2011. URL https://optimization-online.org/?p=11731.

R. Mourad and B. Batut. Optimizing dna sequences for biological functions using a dna llm (galaxy training materials). URL https://training.galaxyproject.org/training-material/topics/statistics/tutorials/genomic-llm-sequence-optimization/tutorial.html. [Online; accessed Sat Nov 29 2025].

J. Mudge, S. Carbonell-Sala, M. Diekhans, et al. Gencode 2025: reference gene annotation for human and mouse. *Nucleic Acids Research*, 53(D1):D966–D975, 11 2024. ISSN 1362-4962. doi:10.1093/nar/gkae1078. URL https://doi.org/10.1093/nar/gkae1078.

L. Nazaretyan, P. Rentzsch, and M. Kircher. varcadd: large sets of standing genetic variation enable genome-wide pathogenicity prediction. *Genome Medicine*, 17(1):84, Aug 2025. ISSN 1756-994X. doi:10.1186/s13073-025-01517-6. URL https://doi.org/10.1186/s13073-025-01517-6.

S. Nurk, S. Koren, A. Rhie, et al. The complete sequence of a human genome. *Science*, 376(6588):44–53, 2022. doi:10.1126/science.abj6987. URL https://www.science.org/doi/abs/10.1126/science.abj6987.

A. Pampari, A. Shcherbina, E. Z. Kvon, et al. Chrombpnet: bias factorized, base-resolution deep learning models of chromatin accessibility reveal cis-regulatory sequence syntax, transcription factor footprints and regulatory variants. *bioRxiv*, 2025. doi:10.1101/2024.12.25.630221. URL https://www.biorxiv.org/content/early/2025/01/08/2024.12.25.630221.

K. S. Pollard, M. J. Hubisz, K. R. Rosenbloom, and A. Siepel. Detection of nonneutral substitution rates on mammalian phylogenies. *Genome Res*, 20(1):110–121, Jan 2010.

e. s. robson and N. Ioannidis. Guanine v1.0: Benchmark datasets for genomic ai sequence-to-function models. In D. A. Knowles and S. Mostafavi, editors, *Proceedings of the 18th Machine Learning in Computational Biology meeting*, volume 240 of *Proceedings of Machine Learning Research*, pages 250–266. PMLR, 30 Nov–01 Dec 2023. URL https://proceedings.mlr.press/v240/robson24a.html.

e. s. robson and N. M. Ioannidis. Enformation theory: A blueprint for evaluating deep learning models in genomics. *bioRxiv*, 2025. doi:10.1101/2024.09.03.611127. URL https://www.biorxiv.org/content/early/2025/01/28/2024.09.03.611127.

P. R. Rosenbaum and D. B. Rubin. The central role of the propensity score in observational studies for causal effects. *Biometrika*, 70(1):41–55, 04 1983. ISSN 0006-3444. doi:10.1093/biomet/70.1.41. URL https://doi.org/10.1093/biomet/70.1.41.

J. F. Salgado. Transforming the area under the normal curve (AUC) into cohen's d, pearson's r pb , Odds-Ratio, and natural log Odds-Ratio: Two conversion tables. *Eur. J. Psychol. Appl. Leg. Context*, 10(1):35–47, 2018.

Y. Schiff, C.-H. Kao, A. Gokaslan, et al. Caduceus: bi-directional equivariant long-range dna sequence modeling. In *Proceedings of the 41st International Conference on Machine Learning*, ICML'24. JMLR.org, 2024.

M. Schubach, T. Maass, L. Nazaretyan, et al. Cadd v1.7: using protein language models, regulatory cnns and other nucleotide-level scores to improve genome-wide variant predictions. *Nucleic Acids Research*, 52(D1):D1143–D1154, 01 2024. ISSN 0305-1048. doi:10.1093/nar/gkad989. URL https://doi.org/10.1093/nar/gkad989.

R. F. Tate. Correlation between a discrete and a continuous variable. point-biserial correlation. *The Annals of Mathematical Statistics*, 25(3):603–607, 1954. ISSN 00034851. URL http://www.jstor.org/stable/2236844.

The 1000 Genomes Project Consortium, et al. A global reference for human genetic variation. *Nature*, 526(7571):68–74, Oct 2015. ISSN 1476-4687. doi:10.1038/nature15393. URL https://doi.org/10.1038/nature15393.

The ENCODE Project Consortium. Expanded encyclopaedias of dna elements in the human and mouse genomes. *Nature*, 583(7818):699–710, Jul 2020. ISSN 1476-4687. doi:10.1038/s41586-020-2493-4. URL https://doi.org/10.1038/s41586-020-2493-4.

The ENCODE Project Consortium, J. Moore, and M. e. a. Purcaro. Expanded encyclopaedias of dna elements in the human and mouse genomes. *Nature*, 583(7818):699–710, Jul 2020. ISSN 1476-4687. doi:10.1038/s41586-020-2493-4. URL https://doi.org/10.1038/s41586-020-2493-4.

E. D. Vaishnav, C. G. de Boer, J. Molinet, et al. The evolution, evolvability and engineering of gene regulatory dna. *Nature*, 603(7901):455–463, Mar 2022. ISSN 1476-4687. doi:10.1038/s41586-022-04506-6. URL https://doi.org/10.1038/s41586-022-04506-6.

xiaoju ye. calflops: a flops and params calculate tool for neural networks in pytorch framework, 2023. URL https://github.com/MrYxJ/calculate-flops.pytorch.

T. Zeng and Y. I. Li. Predicting rna splicing from dna sequence using pangolin. *Genome Biology*, 23(1):103, Apr 2022. ISSN 1474-760X. doi:10.1186/s13059-022-02664-4. URL https://doi.org/10.1186/s13059-022-02664-4.

R. Zhang, P. Isola, and A. A. Efros. Colorful image colorization. In B. Leibe, J. Matas, N. Sebe, and M. Welling, editors, *Computer Vision – ECCV 2016*, pages 649–666, Cham, 2016. Springer International Publishing. ISBN 978-3-319-46487-9.

Z. Zhou, Y. Ji, W. Li, et al. Dnabert-2: Efficient foundation model and benchmark for multi-species genome, 2023.
